# Inhibition of Heat Shock Protein 90 Prevents HIV Rebound*

**DOI:** 10.1074/jbc.M116.717538

**Published:** 2016-03-08

**Authors:** Pheroze Joshi, Ekaterina Maidji, Cheryl A. Stoddart

**Affiliations:** From the Division of Experimental Medicine, Department of Medicine, San Francisco General Hospital, University of California, San Francisco, California 94110

**Keywords:** heat shock protein 90 (Hsp90), human immunodeficiency virus (HIV), hyperthermia, transcription factor, viral transcription, persistent reservoir, viral rebound

## Abstract

HIV evades eradication because transcriptionally dormant proviral genomes persist in long-lived reservoirs of resting CD4^+^ T cells and myeloid cells, which are the source of viral rebound after cessation of antiretroviral therapy. Dormant HIV genomes readily produce infectious virus upon cellular activation because host transcription factors activated specifically by cell stress and heat shock mediate full-length HIV transcription. The molecular chaperone heat shock protein 90 (Hsp90) is overexpressed during heat shock and activates inducible cellular transcription factors. Here we show that heat shock accelerates HIV transcription through induction of Hsp90 activity, which activates essential HIV-specific cellular transcription factors (NF-κB, NFAT, and STAT5), and that inhibition of Hsp90 greatly reduces gene expression mediated by these factors. More importantly, we show that Hsp90 controls virus transcription *in vivo* by specific Hsp90 inhibitors in clinical development, tanespimycin (17-(allylamino)-17-demethoxygeldanamycin) and AUY922, which durably prevented viral rebound in HIV-infected humanized NOD scid IL-2Rγ^−/−^ bone marrow-liver-thymus mice up to 11 weeks after treatment cessation. Despite the absence of rebound viremia, we were able to recover infectious HIV from PBMC with heat shock. Replication-competent virus was detected in spleen cells from these nonviremic Hsp90 inhibitor-treated mice, indicating the presence of a tissue reservoir of persistent infection. Our novel findings provide *in vivo* evidence that inhibition of Hsp90 activity prevents HIV gene expression in replication-competent cellular reservoirs that would typically cause rebound in plasma viremia after antiretroviral therapy cessation. Alternating or supplementing Hsp90 inhibitors with current antiretroviral therapy regimens could conceivably suppress rebound viremia from persistent HIV reservoirs.

## Introduction

Despite complete suppression of plasma viremia by antiretroviral therapy, current drug regimens do not eliminate persistent HIV-producing reservoirs of long-lived resting CD4^+^ T cells and myeloid cells from tissue compartments (1, 2). The viral reservoir in resting cells is established during acute HIV infection, and the integrated provirus remains transcriptionally silent as a result of the absence of activated host transcription factors and cellular epigenetic restrictions (3, 4). Nonetheless, cellular activation can reactivate HIV transcription, and tissue reservoirs are the primary source of viremia rebound within a few weeks after antiretroviral therapy cessation (5, 6).

HIV gene expression is dependent on specific host transcription factors, and cell-activating agents (7, 8), heat shock (9–11), and stress signals (12–14) activate NF-κB, NFAT, STAT5, and P-TEFb required for full-length HIV RNA synthesis (15). These HIV-specific transcription factors are inactive in the cytoplasm of resting cells (16, 17), and in response to cellular activation, the molecular chaperone Hsp90 activates the mature transcription complex (18, 19). Hsp90^2^ is a unique cellular chaperone, and its primary function is post-translational maturation of an exclusive subset of inducible transcription factors, kinases, and steroid hormone receptors. Constitutive Hsp90 expression in unstressed cells maintains cellular homeostasis; however, Hsp90 activity rapidly increases in response to heat shock and activates a specific subset of inducible transcription factors by uncoupling the inhibitory component of the inactive protein complex. These activated transcription factors then translocate into the nucleus and initiate gene expression of vital cellular proteins that protect the cell under stressful conditions (18, 19).

We previously demonstrated that the cytosolic Hsp90 isoform is an essential HIV host factor (20) and showed that heat shock conditions mimicking fever-like hyperthermia (39.5 °C) increased viral replication up to 30-fold in HIV-infected primary human T cells (21). In our original analysis, we confirmed that Hsp90 is a *bona fide* HIV host factor by pharmacologic inhibition and by siRNA-mediated silencing of cellular Hsp90 in primary human cells (20). Hsp90 is a unique member of the heat shock protein family of cellular chaperones in that it uses the energy generated by ATP hydrolysis to activate its client proteins (18, 22, 23). The Hsp90 inhibitors we used (17-(allylamino)-17-demethoxygeldanamycin (17-AAG) and AUY922) have a high affinity for the unique ATP-binding pocket created by Hsp90 dimerization, and these competitive inhibitors specifically block the ATPase activity of the mature Hsp90 protein complex (24). Highly specific second-generation Hsp90 inhibitors currently being evaluated in clinical trials do not interact with other heat shock proteins or cellular factors and have improved bioavailability and significantly reduced toxicity (24, 25).

Heat shock has previously been shown to control HIV reactivation from latency (26), and a recent study suggested that Hsp90 inhibitors prevent HIV gene expression by suppressing NF-κB activation (27). The chaperone function of cellular Hsp90 is not restricted to activating HIV transcription, because we previously demonstrated that replication-incompetent HIV with mutant capsids could be rescued by increased Hsp90 activity (21, 28). We and others also found that Hsp90 is incorporated within the mature virion (21, 29), and there is growing evidence that several virus families exploit cellular Hsp90 for folding and assembly of virus structural proteins and for maturation of viral enzymes (30–32).

Heat shock induces cellular transcription through a rapid increase in Hsp90 activity (33, 34). Previous studies have demonstrated that heat shock increases HIV production and that Hsp90 colocalizes with the site of HIV transcription. In this study, we provide novel evidence that 39.5 °C accelerates transcription from the HIV promoter through specific inducible host transcription factors and that inhibition of Hsp90 greatly reduces gene expression. Inhibition of Hsp90 with specific inhibitors in clinical development, tanespimycin (17-AAG) and AUY922, durably prevented viral rebound in HIV-infected humanized mice even after Hsp90 inhibitor treatment was discontinued. Replication-competent HIV was isolated from the mouse spleens despite undetectable HIV RNA or infected cells in the peripheral blood, indicating the establishment of a persistent tissue reservoir. HIV transcription in the spleen reservoir was reduced by Hsp90 inhibition, but replication-competent virus was readily isolated when the spleen cells were activated by heat shock and by treatment with suberoylanilide hydroxamic acid (SAHA). Here, we present *in vivo* evidence for a persistent HIV-infected tissue reservoir and show that administration of Hsp90 inhibitors for brief periods (2 weeks) prevents rebound in plasma viremia for many weeks after treatment cessation. The ability of Hsp90 inhibitors to suppress HIV transcription *in vivo* was confirmed in chronically infected cell lines, and we demonstrate that Hsp90 inhibition directly affects HIV transcription. Heat shock conditions increased Hsp90 activity in chronically infected cells, and increased virus production at 39.5 °C is the direct result of accelerated HIV transcription.

## Experimental Procedures

### 

#### 

##### Cell Lines, Virus Stocks, and Reagents

HIV-infected 8E5/LAV cells and ACH-2 cells and uninfected Jurkat E6-1 cells were obtained from the National Institutes of Health AIDS Reagent Program (Division of AIDS, NIAID, National Institutes of Health) and were cultured in RPMI 1640 supplemented with 10% fetal bovine serum. Human peripheral blood mononuclear cells (PBMC) were isolated from healthy donors and stimulated with phytohemagglutinin (PHA) for 3 days, and PBMC from six donors were pooled and cryopreserved. The pNL4-3 (35) and pYK-JRCSF (36–38) plasmids were obtained from the AIDS Reagent Program and used to generate infectious virus stocks by transfecting HEK 293T cells. The virus titer in the infectious culture supernatant was determined in PHA-stimulated PBMC by end point dilution with assessment of supernatant HIV p24 by ELISA (PerkinElmer Life Sciences) after 7 days, and 50% tissue culture infectious doses were calculated using the Reed-Muench method.

The following cell culture grade reagents were purchased from Sigma: 5′-iodo-2′-deoxyuridine (IUdR), cycloheximide, brefeldin A, tunicamycin, cytochalasin D, wortmannin, and human tumor necrosis factor-α (TNF-α). Thapsigargin and jasplakinolide were purchased from Abcam. 4′-Ethynyl-2-fluoro-2′-deoxyadenosine (EFdA) was provided by Yamasa Corp. (Chiba, Japan) and also obtained by custom synthesis (Life Chemicals Inc., Burlington, Canada). The Hsp90 inhibitor 17-AAG (tanespimycin) was purchased from Sigma, and NVP-AUY922 (AUY922) was purchased from Selleckchem. SAHA was obtained from the NIH AIDS Reagent Program.

##### Dual-Glo Luciferase Reporter Assay

To measure the effect of 39.5 °C on HIV transcription, we used the Dual-Glo luciferase reporter assay system from Promega. The individual eukaryotic transcription factor binding sequences found in the HIV U3 promoter were purchased from Promega as independent luciferase reporter vectors and included AP1 (pGL4.44[luc2P/AP1-RE/Hygro]), NF-κB (pGL4.32[luc2P/NF-κB-RE/Hygro]), NFAT (pGL4.30[luc2P/NFAT-RE/Hygro]), and STAT5 (pGL4.52[luc2P/STAT5-RE/Hygro]). The entire HIV U3 region from the 5′-LTR was PCR-amplified from pNL4-3 and cloned into the pGL4.44[luc2P/AP1-RE/Hygro] vector by replacing the AP1 binding sequence. To ensure equal transfection efficiency, sample loading, and assay conditions, we modified the *Renilla reniformis* loading control vector (pGL4.84[*hRluc*CP/Puro] vector) and used the translation elongation factor 1A (EF1α) promoter sequence to drive *Renilla* luciferase gene expression. 10 μg of plasmid DNA of each luciferase reporter construct was mixed with 1 μg of control EF1α/*hRluc* plasmid DNA and independently transfected in 1 × 10^6^ 8E5/LAV cells and Jurkat E6-1 cells. The transfected cultures were incubated for 12 h at 37 °C, cells were washed in PBS and resuspended in fresh medium, and the cell culture was evenly split in 96-well tissue culture plates and incubated at 37, 39.5, and 39.5 °C with 1 μm 17-AAG for 8–16 h, depending on the individual promoter sequence activity, as recommended by Promega. Following the prescribed incubation, the Dual-Glo firefly luciferase substrate (beetle luciferin) was added directly into the culture supernatant and incubated at room temperature for 10 min, and luminescence was measured in a white-coated 96-well plate on a M2 SpectraMax plate reader (Molecular Devices) with normal speed and medium photomultiplier tube settings. Immediately after, the Dual-Glo Stop & Glo *Renilla* luciferase substrate (coelenterazine) was added to the wells and incubated at room temperature for 10 min, and luminescence was measured with normal speed and medium photomultiplier tube settings.

##### Ethics Statement

Mice were housed in a barrier facility staffed and maintained by the University of California San Francisco Laboratory Animal Resource Center under Public Health Service Assurance of Compliance A3400-01. The University of California San Francisco is accredited by the Association for Assessment and Accreditation of Laboratory Animal Care International. All animal experiments were conducted in accordance with the Guide for the Care and Use of Laboratory Animals and approved by the University of California San Francisco institutional animal care and use committee.

##### NSG-BLT Mice

Mice were housed in a barrier facility in individually ventilated plastic cages (Innovive Innorack IVC) with corn cob bedding and a maximum of 5 mice/cage. Mice were provided with environmental enrichment (nesting material), and lighting was timer regulated with 12 h on/12 h off. Humidity was 30–70%, temperature was 68–79 °F, and food and water were provided *ad libitum*. Mice were observed at the time of dosing every day and were weighed every 2–5 days. Two cohorts of NSG-BLT mice were generated by implanting 8–10-week-old female (mean weight: 26 g) NOD-scid IL-2Rγ^−/−^ (NSG) mice (stock no. 005557, NOD.Cg-*Prkdc^scid^ Il2rg^tm1Wjl^*/SzJ, Jackson Laboratory) under the kidney capsule with 1-mm^3^ pieces of human fetal thymus and liver from a single donor for each cohort, as described previously (39, 40). Three weeks after implantation, mice were injected intravenously with 200,000 viable human CD45^+^ CD34^+^ Lin-1^−^ hematopoietic stem/progenitor cells isolated from the autologous fetal liver with a CD34 positive selection kit (StemCell Technologies) and cryopreserved. The mice were not irradiated prior to cell injection because we have found that this conditioning step is not necessary for robust human leukocyte reconstitution.

##### Humanized Mouse Study Design and Experimental Procedures

Two mouse experiments with 30 mice each were performed, each with four groups of mice (5–9 mice/group) that were assigned to treatment groups in a completely randomized design. Mice were numbered to blind technicians from sample treatment identity. The number of mice per group was calculated to provide statistical power sufficient to show that a decrease of 0.5 log_10_ copies of HIV RNA resulting from antiviral drug treatment is statistically significant by the Mann-Whitney *U* test. The second mouse experiment was an independent replication of the first experiment.

NSG-BLT mice were anesthetized with isoflurane and inoculated intraperitoneally with 200 μl of virus stock containing 25,000 50% tissue culture infectious doses of HIV_JR-CSF_ through an intravenous catheter (BD Insyte, catalog no. 381512) and a 1-ml tuberculin syringe. Mice were inoculated 8–9 weeks after CD34^+^ cell injection and were treated with EFdA and/or Hsp90 inhibitors beginning 5 weeks (Fig. 2*A*) or 2 weeks (Fig. 4*A*) after HIV inoculation. EFdA (10 mg/kg/day in PBS) was administered by once-daily oral gavage (of 200 μl), and 17-AAG and AUY922 (both 10 mg/kg/day in water) were administered by twice-daily subcutaneous injection (of 200 μl). AUY922 was dissolved in 100% ethanol before dilution in water. Mice were anesthetized with isoflurane, and 200 μl of peripheral blood was collected from the retroorbital sinus with a glass Pasteur pipette into a blood collection tube containing EDTA (BD Biosciences, catalog no. 365974). For terminal blood collection, mice were anesthetized with isoflurane, and blood was collected from the severed right brachial artery and vein with a transfer pipette flushed with 10% EDTA into blood collection tubes containing EDTA (Sarstedt, product no. 41.1395.105). Each mouse was then euthanized by cervical dislocation. For HIV RNA quantification by branched DNA assay, mouse plasma was lysed with reagents supplied by the manufacturer (VERSANT^TM^ HIV-1 RNA 3.0 Assay, Siemens). The limit of detection was 75 copies/100 μl.

##### Flow Cytometry

Human leukocyte reconstitution was assessed by flow cytometry using Trucount tube (BD Biosciences) enumeration to calculate the absolute numbers of human leukocytes, including CD4^+^ T cells, per μl of blood (41). Anti-human CD45-Alexa 700 (Caltag) and anti-mouse CD45-allophycocyanin (BD Biosciences) antibodies were used to differentiate mouse from human leukocytes, and human CD45^+^ cells were typed using antibodies specific for human leukocyte markers, including CD3-ECD and CD4-Pacific Blue.

##### Infectious Units per Million Cells (IUPM) Assay

We used a modified IUPM assay to determine the replication capacity of infected cells isolated from the peripheral blood and spleens of humanized mice. The IUPM assay was modified for the detection of low levels of replication-competent virus that could infect and spread in physiologically relevant target cells. To track viremia during the course of Hsp90 inhibitor treatment, we used a spreading infection end point dilution format wherein the cells were diluted in half-log increments from 100,000 to 32 cells/well. The diluted cells were incubated with 10^5^ PHA-stimulated normal human PBMC for 7 days, and wells were scored as positive by detection of HIV p24 antigen in the assay supernatant. The IUPM was calculated by the Reed-Muench method.

##### Effect of 17-AAG Pretreatment on HIV Replication

We pretreated PHA-stimulated PBMC with 1 μm 17-AAG for 24 h, washed the cells, and inoculated equal numbers of cells with a multiplicity of infection of 0.1 and 0.001 of HIV_NL4-3_ from day 1 to day 6. At each time point, the pretreated cells were inoculated with virus for 2 h, washed in complete medium to remove residual virus, and cultured for an additional 7 days when supernatant p24 was detected by ELISA. Parallel cellular cytotoxicity of the drug was determined on 17-AAG-pretreated PBMC by the MTT assay as described below.

##### In Vitro Antiviral Activity Assay

The antiviral activity of 17-AAG and AUY922 was assayed in PHA-stimulated PBMC as described previously (42). Briefly, PBMC were inoculated in bulk at a multiplicity of infection of 0.001 of HIV_NL4-3_ for 2 h, cells were washed, and 1 × 10^5^ cells in 100 μl were seeded in triplicate wells of a 96-well plate. Wells were treated with 100 μl of serial half-log dilutions of 17-AAG or AUY922 or with medium alone, and culture supernatants were assayed at day 7 for p24 by ELISA. Parallel cellular toxicity of the drug was determined on uninfected cells by a 3-(4,5-dimethylthiazol-2-yl)-2,5-diphenyl-tetrazolium bromide (MTT) assay (Sigma) on day 7. The 50% inhibition of virus replication (IC_50_) and 50% reduction in cell viability (CC_50_) were calculated as the mean of three independent assays.

##### Cell-associated HIV DNA Copy Number and Human CCR5 Copy Number

Total cellular DNA was extracted from equivalent cell numbers from each assay using the DNA blood minikit (Qiagen, GmbH) and eluted in 100 μl. Mouse peripheral blood cells and spleen cells were treated with ammonium-chloride-potassium (ACK) lysis buffer to remove erythrocytes prior to DNA extraction. HIV DNA copy number was estimated with the TaqMan fast advanced master mix (Applied Biosystems) using the same 5′-LTR primer-probe combination as described below. To report the HIV DNA copy number per 10^6^ human cells, the extracted DNA was used to detect genomic CCR5 copies (forward primer, 5′-GTTGGACCAAGCTATGCAGGT-3′; reverse primer, 5′-AGAAGCGTTTGGCAATGTGC-3; internal probe, 5′-/56FAM/TTGGGATGA/ZEN/CGCACTGCTGCATCAACCCCA/3IABkFQ/-3′). The quantitative PCR used 5 μl of DNA extract with 150 nm forward and reverse primers and 41.4 nm probe in a total 50-μl reaction volume (43), and reactions were run on a StepOnePlus^TM^ real-time PCR system (Applied Biosystems).

##### RNAscope and Immunohistochemistry

Spleen samples obtained from BLT mice were fixed in 3.7% formaldehyde and infiltrated with 5–15% sucrose, followed by embedding in optimal cutting temperature compound and freezing in liquid nitrogen. We used a novel next generation *in situ* hybridization technique (RNAscope) developed by Advanced Cell Diagnostics. This technology utilizes a unique “double Z” probe design, which greatly increases signal/noise ratio with visualization of single RNA transcripts. RNAscope for HIV RNA was performed using the 2.0 HD Reagent Kit-RED (catalog no. 310036) according to the manufacturer's instructions. HIV RNA was detected using a HIV-gag-pol probe (catalog no. 317691). To confirm the specificity of *in situ* hybridization, we used spleen samples from control NSG mice as a negative control. Human peptidyl-prolyl *cis-trans*-isomerase B (PPIB) encoded by the *PPIB* gene was detected with the Hs-PPIB probe in the HeLa cell control (catalog nos. 313901 and 310045) and served as an RNAscope positive control. The RNAscope assay was followed by standard immunohistochemistry for HIV p24 with mouse mAb anti-HIV-1 p24 from the AIDS Reagent Program as the primary antibody (catalog no. 3537) and polymeric HRP-linked goat anti-mouse IgG secondary antibody detected using the DAB-Ni substrate kit (catalog nos. D37-110 and C10-12, Golden Bridge International). Nuclei were counterstained with hematoxylin QS (catalog no. H-3404, Vector Laboratories). Tissue sections were analyzed on a Leica DM6000 B microscope equipped with an HCX PL FLUOTAR ×63/0.90 CORR air immersion objective and Leica DFC 500 camera. Bright field images were acquired at a total optical magnification of ×630 (18 °C) with LAS version 4.3 software. ImageJ software was used to digitally quantify HIV RNA^+^ cells.

##### Quantification of 8E5/LAV Virus Production at 39.5 °C

8E5/LAV cells were grown at 37 °C for 24 h, washed three times with PBS to remove any residual virions, and resuspended in fresh medium. The cell culture was divided evenly, 1 × 10^6^ 8E5/LAV cells were incubated at 37 and 39.5 °C with and without 100 μg/ml IUdR for 24 h, and the culture supernatant was assayed for 8E5/LAV viral RNA by quantitative PCR as described below and for HIV p24 by ELISA. To determine the effect of cycloheximide on virion production, actively growing 8E5/LAV cells were cultured and washed as described above. Equal cell numbers were seeded into 96-well plates in the presence of 10 μg/ml cycloheximide, the plates were incubated at 39.5 °C for 12 h, supernatant p24 was measured by ELISA, and cell-associated 8E5/LAV RNA was measured by quantitative PCR as described below.

##### Cell-free and Cell-associated 8E5/LAV and ACH-2 HIV RNA Copy Number

The 8E5/LAV or ACH-2 viral RNA copy number in the culture supernatants was calculated with the Abbott RealTime HIV-1 test using 0.6 ml of supernatant and standard input volume for a lower limit of detection of 40 copies/ml. Briefly, viral RNA was extracted from 0.6 ml of 8E5/LAV or ACH-2 supernatant using the QIAmp MiniElute virus spin kit (Qiagen, GmbH) along with the Qiagen RNase-free DNase kit, and total RNA was eluted in 50 μl. Purified HIV RNA copy number was estimated by quantitative reverse transcription PCR (44) using the RNA-to-CT 1-Step TaqMan quantitative PCR system (Life Technologies, Inc.), and reactions were run on the StepOnePlus^TM^ real-time PCR system (Applied Biosystems). HIV-specific primers (200 nm each) and probe (200 nm) against the 5′-LTR (forward primer, 5′-GCCTCAATAAAGCTTGCCTTGA-3′; reverse primer, 5′-GGGCGCCACTGCTAGAGA-3′; internal probe, 5′-CCAGAGTCACACAACAGACGGGCACA-3′ FAM-BQ) were used for both the test assay samples and the standard curve. Cell-associated HIV RNA copy number was estimated on total RNA extracted from an equal number of cells using the QIAmp MiniElute virus spin kit (Qiagen, GmbH) along with the Qiagen RNase-free DNase kit as described above and eluted in 50 μl. Cell-associated HIV RNA copy number was estimated using the RT-PCR conditions and primers as described above, and the copy number was reported as HIV RNA copies/10^6^ 8E5/LAV or ACH-2 cells.

##### Cell Status at 39.5 °C

The effect of 39.5 °C on cell viability and proliferation was determined using the LIVE/DEAD fixable dead cell stain (Life Technologies), and the green fluorescent reactive dye was measured by flow cytometry (BD Biosciences). The effect of 39.5 °C on cellularity was determined by intracellular acetylcholinesterase activity (45, 46). Briefly, 10^5^ cells were resuspended in 100 μl of PBS and mixed with 100 μl of 1.25 mm acetylthiocholine (pH 8) and 0.1 mm 5,5-dithio-bis(2-nitrobenzoic acid) (pH 7) in PBS. The incubation was performed at room temperature, but the test samples and the substrate solution were prewarmed to 37 °C for 10 min before reading the optical density. Changes in absorption were monitored at 450 nm for 10 min with a plate reader spectrophotometer. The sensitivity of the assay was determined using serial dilutions of *Electrophorus electricus* acetylcholinesterase (Sigma), and acetylcholinesterase enzyme kinetics (*V*_max_) were calculated using the Michaelis-Menten method.

##### Western Blotting Analysis

To detect HIV proteins, the culture supernatant was clarified by low speed centrifugation and filtered through a 0.22-μm membrane, and virions were sedimented through a 20% sucrose cushion at 100,000 × *g* for 2 h at 4 °C. The virus pellet was resuspended in PBS and overlaid on 2 ml of 8.4% iodixanol (OptiPrep, Sigma). HIV virions were sedimented at 100,000 × *g* for 1.5 h and resuspended in PBS at 10 μg of p24/ml. The equivalent of 100 ng of p24 of purified virus was separated on a 8–20% linear gradient polyacrylamide gel, electroblotted onto Immobilon PVDF membranes (Millipore), and reacted individually with the following primary antibodies obtained from the AIDS Reagent Program: anti-HIV immunoglobulin (catalog no. 3957, lot 130230) prepared from pooled plasma of asymptomatic, HIV antibody-positive donors with CD4^+^ T-cell counts above 400/μl; mouse monoclonal antibody against HIV-1 p24 (catalog no. 6521, lot 100098) affinity-purified from ascites fluid; and HIV-1 gp120 mouse monoclonal antibody (ID6) (catalog no. 2343, lot 060840) raised against a recombinant LAV-1 gp160 preparation. ID6 is an IgG2a antibody that binds to gp120 and gp160 and is directed against the first 204 amino acids of gp120, and HIV-1 gp41 monoclonal antibody (F240) (catalog no. 7623, lot 120129) was obtained from an EBV-transformed heteromyeloma. Rabbit anti-human IgG H&L (catalog no. 6759) and goat anti-mouse IgG (catalog no. 7068) horseradish peroxidase-conjugated secondary antibodies were purchased from Abcam, and immune complexes were visualized by enhanced chemiluminescence (Pierce). To quantify cellular Hsp90 and loading controls, cell lines were lysed in CelLytic M solution (Sigma), and total protein concentration was estimated using bicinchoninic acid (Sigma). Whole-cell extract (10 μg) was resolved on a stacked 12% denaturing polyacrylamide gel, electroblotted, and probed using the following primary antibodies from Abcam: rabbit anti-Hsp90 (catalog no. ab109704), rabbit anti-actin (catalog no. ab1801), and mouse anti-GAPDH (catalog no. ab9484). Protein concentrations were quantified using ECL Plex goat anti-mouse IgG-Cy5 (catalog no. PA43010) or goat anti-rabbit IgG-Cy5 (catalog no. PA45012) secondary antibodies (GE Healthcare), and the fluorescent intensity was measured on a Typhoon Trio variable mode imager (GE Healthcare). Immunoblots were scanned at 200-μm resolution with a 450 photomultiplier tube setting, and band intensities were calculated using ImageQuant version 7 software (GE Healthcare).

Prior to selecting housekeeping targets as protein loading controls, we determined whether cell culture conditions at 39.5 °C affected actin and GAPDH expression levels. Cells growing in a continuous culture were evenly divided and incubated at 37 and 39.5 °C for up to 48 h. At each time point, cell suspensions were directly lysed in 2× Laemmli buffer (Sigma), and equal volumes of the lysate were loaded in adjacent wells of a 12% denaturing polyacrylamide gel. In addition, equal numbers of cells were lysed in CelLytic M solution and quantified, and increasing concentrations (0.5–10 μg) were assayed for both loading controls. We confirmed that cell culture conditions at 39.5 °C did not affect the expression levels of both actin and GAPDH over multiple time points and protein concentrations.

##### Nitric-oxide Synthase Assay

A standard nitric-oxide synthase assay was used as an indicator of Hsp90 activity at 39.5 °C (47), wherein nitric-oxide synthase converts arginine into l-citrulline and nitric oxide (NO). The NO reacts with the amino groups in 4,5-diaminofluorescein (Sigma) and converts the substrate into fluorescent triazolofluorescein. Briefly, 8E5/LAV and ACH-2 cells were incubated up to 14 h with sampling every 2 h, and 10^5^ cells were washed in PBS and seeded in black, clear bottom, 96-well plates in reaction buffer containing 100 μm l-arginine and 0.1 μm 4,5-diaminofluorescein and incubated for 5 min in the dark at room temperature. Fluorescence was measured at room temperature using a spectrofluorometer (SpectraMax M2, Molecular Devices) with an excitation wavelength at 495 nm and emission wavelength at 515 nm. The bandwidth was 10 nm for both excitation and emission, and the sensitivity was programmed to high. Thapsigargin was used at 10 μm as a positive control for nitric-oxide synthase activity.

##### Antiviral Activity of 17-AAG on 8E5/LAV Virus Production

Actively growing 8E5/LAV cells and 8E5/LAV cells pretreated with 100 μg/ml of IUdR were seeded into 96-well tissue culture plates with half-log dilutions of 17-AAG and then incubated at either 37 or 39.5 °C for 24–48 h with supernatant p24 determined by ELISA. Cytotoxicity was measured using the MTT assay as described above. Cell-associated RNA was extracted from an equal number of cells, and copy number/10^6^ cells was estimated as described above.

##### Denatured Luciferase Assay

To determine whether 17-AAG was biologically active and retained anti-Hsp90 activity at 39.5 °C, we used a standard denatured luciferase assay (48). Briefly, 250 nm firefly luciferase (Sigma) in denaturation buffer (25 mm Tris, pH 7.5, 8 mm MgSO_4_, 0.01% bovine γ-globulin, 10% glycerol) was incubated at 50 °C for 8 min, and 5-μl aliquots were cooled to room temperature for 3 min and then diluted to 25 μl in renaturation buffer (25 mm Tris, pH 7.5, 8 mm MgSO_4_, 0.01% bovine γ-globulin, 10% glycerol, 0.5 mm ATP, 5 mm KCl, 2 mm DTT). The 8E5/LAV cells at 37 and 39.5 °C, with or without 17-AAG, were gently lysed in the luciferase assay lysis buffer, and 25 μl was added to the heat-denatured luciferase reaction. The renaturation reaction was carried out at room temperature for 1 h, and 5 μl of the reaction was then added to 45 μl of the Steady-Glo luciferase substrate (Promega) in a white-coated 96-well plate with luminescence measured on an M2 SpectraMax plate reader (Molecular Devices) with normal speed and medium photomultiplier tube settings. Controls were performed identically in the absence of ATP and the renaturation step.

##### Selective Inhibition of 8E5/LAV Production

Before treatment with the following agents, 8E5/LAV cultures were grown for 24 h at 37 °C. The cells were washed three times with PBS to remove residual virus particles and resuspended in fresh medium with the indicated test reagent, and the cell cultures were split evenly and incubated at 37 °C with 100 μg/ml IUdR or at 39.5 °C for the specified duration. 8E5/LAV cells were incubated with 10 μg/ml brefeldin A for 6 h, virus-like particles in the culture supernatant were assayed for viral p24, and cell-free virus-like particles were purified and subjected to immunoblotting to detect gp120 and gp41. 8E5/LAV cells were incubated with 1 or 5 μg/ml tunicamycin for 8 h, and the cells were washed in PBS and lysed in radioimmune precipitation assay buffer. Twenty μg of total cell extracted protein was subjected to immunoblotting to detect intracellular gp120. To study the effect of 39.5 °C on virion transport and egress, we incubated 8E5/LAV cells in the presence of half-log dilutions of cytochalasin D (starting concentration 100 μg/ml), jasplakinolide (starting concentration 100 mm), and wortmannin (starting concentration 100 mm). Cultures were maintained at the respective temperature for 6 h, the supernatants were assayed for virion p24, and cells were either lysed for detection of cell-associated HIV RNA or subjected to the MTT cytotoxicity assay as described elsewhere.

##### TZM-bl Assay with ACH-2 HIV

To determine whether 39.5 °C affected the infectivity of the ACH-2 virus in the culture supernatant, we used the TZM-bl dual reporter cell line. ACH-2 cells were harvested from an actively growing culture; washed in PBS to remove any residual virions attached to the cell membrane; evenly split in fresh culture medium; and incubated at 39.5 °C in the presence of cycloheximide (10 μg/ml), brefeldin A (10 μg/ml), tunicamycin (1 μg/ml), cytochalasin D (10 ng/ml), jasplakinolide (1 μm), and wortmannin (1 μm). The cultures were incubated for 8 h, after which an equal volume of supernatant was added to adherent TZM-bl cells and incubated for 48 h at 37 °C. TZM-bl cells were washed in PBS; the Bright-Glo luciferase substrate and lysis buffer (Promega) were added directly to the wells; and, following a 2-min incubation, cell lysates were transferred to a white-coated 96-well plate, and luminescence was measured with the spectrophotometer settings described above.

##### Statistics

**FIGURE 1.**
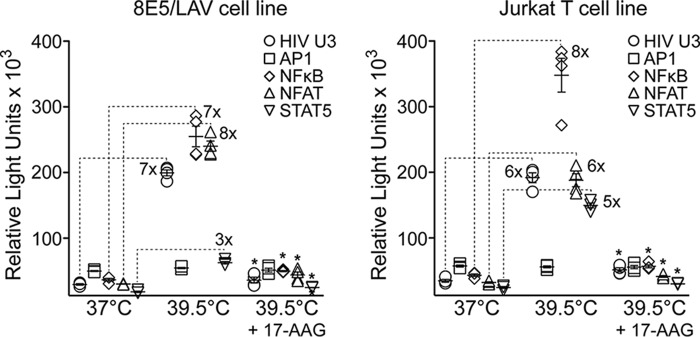
**Hsp90 controls HIV transcription.** Shown is the Dual-Glo luciferase assay at 37 and 39.5 °C and at 39.5 °C with 17-AAG in HIV-infected 8E5/LAV cells (*left*) and in uninfected Jurkat T cells (*right*). Data represent mean ± S.E. (*error bars*) (*n* = 4). *, *p* < 0.0001 by Student's *t* test comparing luciferase expression at 39.5 °C with luciferase expression at 39.5 °C in the presence of 1 μm 17-AAG.

**FIGURE 2.**
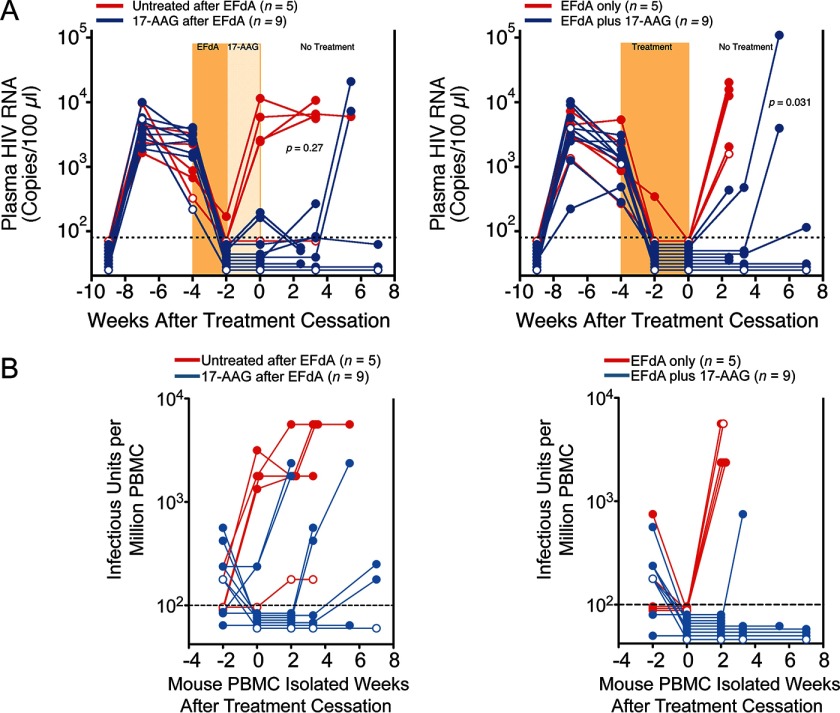
**Hsp90 inhibition prevents virus rebound for several weeks after treatment cessation.** *A*, groups of viremic HIV_JR-CSF_-infected NSG-BLT mice were treated orally once a day with 10 mg/kg/day EFdA for 2 weeks (*left*) or 4 weeks (*right*). Separate groups were treated by twice-daily subcutaneous injection of 17-AAG (10 mg/kg/day) for 2 weeks either after EFdA (*left*) or concurrent with EFdA for the last 2 weeks (*right*). The treatment period is indicated by *shading*, and mice in both comparisons were from the same NSG-BLT mouse cohort. Mouse peripheral blood human CD4^+^ T cell counts at the time of treatment cessation were highly similar between groups with means of 100 CD4^+^ T cells/μl (untreated after EFdA), 210 CD4^+^ T cells/μl (17-AAG after EFdA), 130 CD4^+^ T cells/μl (EFdA only), and 130 CD4^+^ T cells/μl (EFdA plus 17-AAG). *p* values for Fisher's exact test for rebounders *versus* nonrebounders were determined at the last blood collection. *B*, IUPM assay on mouse PBMC samples from mice treated with EFdA followed by 17-AAG (*left*) and mice treated simultaneously with EFdA and 17-AAG for the experiment shown in Fig. 6*A*. Sample testing began 2 weeks before treatment cessation and continued until mouse termination. *Open circles*, mice that were not injected with CD34^+^ hematopoietic stem/progenitor cells. Data for EFdA-only control mice in *A* were published previously (46).

*p* values were calculated by Student's *t* test (Figs. 1, 6, 7, and 9), Fisher's exact test (Figs. 2*A* and 4*A*), and the Mann-Whitney *U* test (Fig. 5*B*). The unit of analysis was a single mouse.

## Results

### 

#### 

##### Transactivation of the HIV Promoter at 39.5 °C Is Dependent on Hsp90 Activity

The conditionally active cellular transcription factors NF-κB (49, 50), NFAT (51), cyclin T1/CDK9 (52), and STAT5 (53) are activated exclusively by Hsp90 and are essential for the initiation and elongation of HIV transcription (54). The DNA recognition sequences for these host transcription factors are located within the U3 region of the HIV promoter in the 5′-LTR of the integrated proviral genome. To determine whether 39.5 °C accelerates transcription from the HIV U3 promoter and whether the individual host transcription factors are activated at 39.5 °C, we used the Dual-Glo firefly/*Renilla* luciferase assay system in the chronically infected 8E5/LAV cell line (55). The 8E5/LAV cell line has a single integrated HIV genome that requires stimulation to produce virus. This genetic reporter system comprises independent cellular transcription factor recognition sequences cloned upstream of the luciferase reporter. We initially tested the entire HIV U3 region and observed a 7-fold increase in luciferase gene expression at 39.5 °C (Fig. 1). We then determined whether the HIV-specific host transcription factors could independently accelerate luciferase gene expression at 39.5 °C and whether inhibition of Hsp90 activity with the Hsp90 inhibitor tanespimycin (17-AAG) (25) at 39.5 °C decreased transcription of the luciferase reporter. Using the pGL4 luciferase reporter vectors (Promega), we found that 39.5 °C substantially increased luciferase gene expression by NF-κB (7-fold), NFAT (8-fold), and STAT5 (3-fold) transcription factors in 8E5/LAV cells. Interestingly, gene expression by the constitutively expressed AP1 transcription factor, which is not activated by Hsp90, was unaffected at 39.5 °C, and importantly, 17-AAG significantly reduced (*p* < 0.0001 compared with no drug treatment at 39.5 °C) HIV U3-, NF-κB-, NFAT-, and STAT5-mediated luciferase gene expression at 39.5 °C. Similar gene expression profiles at 39.5 °C were observed in the uninfected Jurkat T-cell line (Fig. 1). We modified a loading control vector with the EF1α promoter sequence to drive *Renilla* luciferase expression and confirmed that 17-AAG treatment at 39.5 °C did not affect any of the assay parameters (data not shown).

##### Hsp90 Inhibition Prevents Rebounds in HIV Viremia

Our *in vitro* findings provided compelling evidence for the key role of Hsp90 in HIV persistence and reactivation, so we proceeded to test our hypothesis *in vivo* by administering Hsp90 inhibitors to HIV-infected humanized mice with fully suppressed plasma viremia (42). To achieve complete viral suppression, we treated HIV_JR-CSF_-infected NSG-BLT mice with the ultrapotent (50 pm IC_50_) nucleoside analog reverse transcriptase inhibitor EFdA (56) for 2 or 4 weeks by once-daily oral gavage of 10 mg/kg/day (42). We performed two independent studies in healthy drug-naive mice; the mean mouse weight for the first experiment was 26.9 g, and the mean weight for the second experiment was 26.7 g.

In our first study, we chose two different regimens: suppression of viremia with EFdA for 2 weeks before 17-AAG administration (Fig. 2*A*, *left*) or suppression with EFdA for 4 weeks concurrent with 17-AAG administration for the last 2 weeks (Fig. 2*A*, *right*). In mice treated with EFdA alone for 2 weeks (Fig. 2*A*, *left*) or 4 weeks (Fig. 2*A*, *right*), viral rebound to pretherapy levels occurred in 9 of 10 (90%) mice within 2–3 weeks of treatment cessation. In stark contrast, only 1 of 9 mice (11%) treated with 17-AAG after EFdA suppression (Fig. 2*A*, *left*) and 2 of 9 mice (22%) treated with 17-AAG during EFdA suppression (Fig. 2*A*, *right*) had evidence of viral rebound 3 weeks after treatment cessation. No adverse events were observed in any of the treated mice. Four additional 17-AAG-treated mice rebounded by 5 weeks after treatment cessation, but at 7 weeks, 6 of the 18 mice still had no detectable plasma HIV RNA (<75 copies/100 μl). Importantly, 17-AAG exerted this effect in the mice while preserving their peripheral blood human CD4^+^ T-cell counts, so the lack of viral rebound was not the result of loss of HIV target cells. The inhibition of viral rebound in the mice resulting from Hsp90 inhibition was also demonstrated by a sensitive IUPM virus outgrowth assay that quantifies the number of cells with replication-competent HIV in the peripheral blood (Fig. 2*B*).

##### Effects of Hsp90 Inhibition Can Persist after Removal of the Drug

**FIGURE 3.**
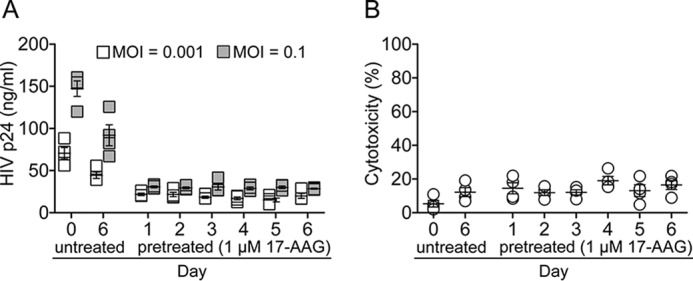
**Long lasting effect of Hsp90 inhibition.** *A*, PBMC pretreated with 1 μm 17-AAG for 24 h were inoculated with HIV at multiplicity of infection of 0.001 and 0.1 from 1 to 6 days after drug treatment. Supernatant p24 was estimated after 7 days of culture. *B*, uninfected PBMC pretreated with 1 μm 17-AAG for 24 h were tested for cytotoxicity from 1 to 6 days after drug treatment. Data represent mean ± S.E. (*error bars*) (*n* = 4).

The long term pharmacodynamic effects of Hsp90 inhibitors have been reported in preclinical studies with primary cells, where the effect of a single dose lasted more than 72 h after drug treatment (57, 58). Further, the long term *in vivo* effects of 17-(dimethylaminoethylamino)-17-demethoxygeldanamycin) (17-DMAG), a synthetic derivative of 17-AAG, were observed as long as 14 weeks after treatment cessation (59). To determine the long term effects of Hsp90 inhibition, we pretreated human PBMC with 17-AAG for 24 h and then inoculated the cells with a range of multiplicities of infection beginning 1–6 days after treatment (Fig. 3*A*). The antiviral effect of 17-AAG was maintained for 6 days after treatment with no cellular cytotoxicity (Fig. 3*B*). Untreated PBMC were susceptible to HIV infection after 6 days in culture, thus indicating that 17-AAG pretreatment can inhibit HIV replication for nearly 1 week after drug treatment.

##### Hsp90 Inhibition Prevents HIV Transcription in Persistent Viral Reservoirs

**FIGURE 4.**
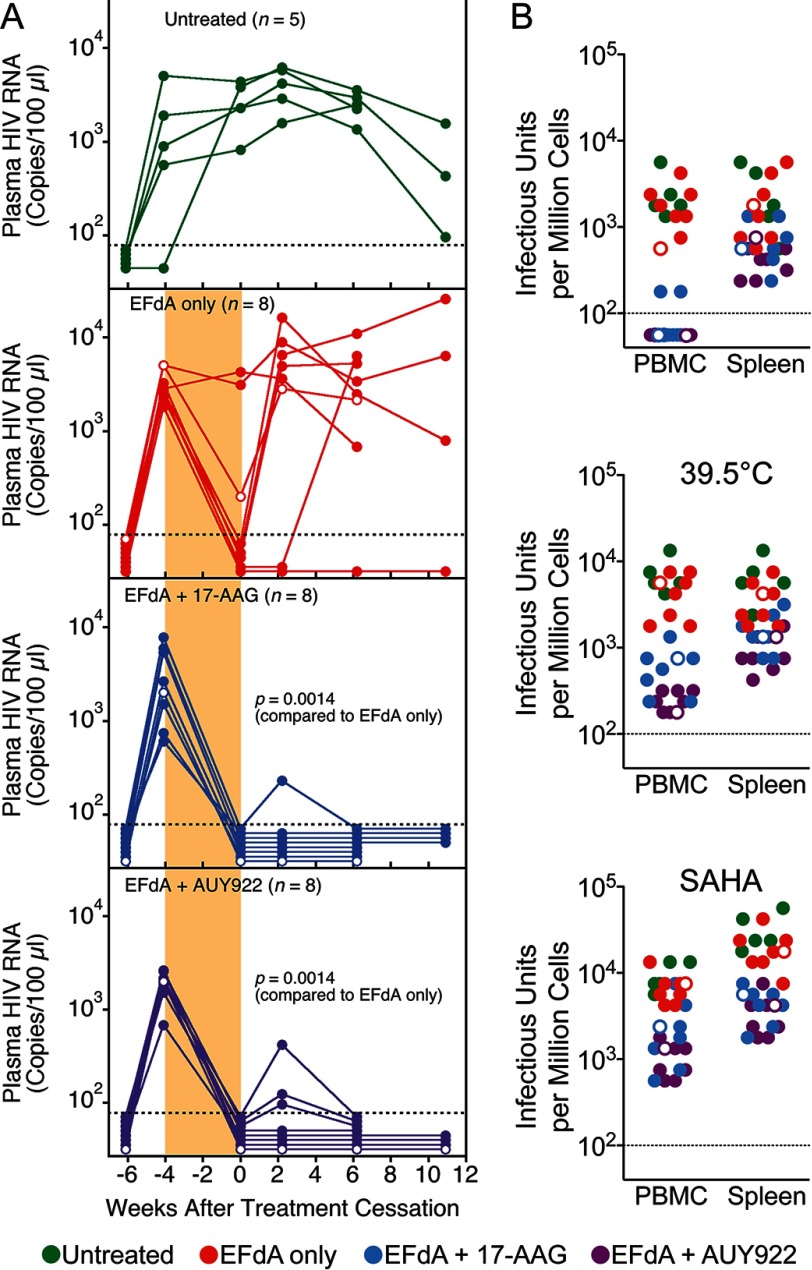
**Hsp90 inhibition reduced the persistent tissue reservoir.** *A*, repeat experiment with AUY922, a second-generation Hsp90 inhibitor. Groups of viremic HIV_JR-CSF_-infected NSG-BLT mice were treated orally once a day with 10 mg/kg/day EFdA only for 4 weeks or with EFdA plus twice-daily subcutaneous injection of 17-AAG or AUY922 (10 mg/kg/day) for the last 2 weeks. The treatment period is indicated by *shading*, and all mice were from the same NSG BLT mouse cohort. Mouse peripheral blood human CD4^+^ T cell counts at the time of treatment cessation were highly similar between groups with means of 130 CD4^+^ T cells/ μl (untreated), 110 CD4^+^ T cells/μl (EFdA only), 94 CD4^+^ T cells/μl (EFdA plus 17-AAG), and 66 CD4^+^ T cells/μl (EFdA plus AUY922). *Open circles*, implanted mice in the cohort that were not injected with CD34^+^ hematopoietic stem/progenitor cells. *p* values for Fisher's exact test are shown. *B*, IUPM assay on terminal PBMC and spleen cells (obtained at both 6 and 11 weeks after treatment cessation from mice shown in *A*) at 37 and 39.5 °C and in the presence of 10 nm SAHA. Serial dilutions of mouse-derived cells were cocultivated with PHA-stimulated PMBC for 7 days, and IUPM were determined by detection of p24 in the supernatants.

To confirm and extend our *in vivo* findings, we performed a second NSG-BLT mouse study in which we compared 17-AAG with AUY922 (24, 60), a second-generation Hsp90 inhibitor with more potent, >3.5-fold anti-HIV activity *in vitro*. The antiviral activity of AUY922 (IC_50_ = 0.03 μm) and 17-AAG (IC_50_ = 0.11 μm, *p* = 0.03) were assayed in human PBMC, and both Hsp90 inhibitors had similar low level cytotoxicity (AUY922 CC_50_ = 1.24 μm and 17-AAG CC_50_ = 0.95 μm). As we observed in the first mouse study, treatment of HIV_JR-CSF_-infected NSG-BLT mice with EFdA plus 17-AAG prevented sustained rebounds in plasma HIV RNA up to 11 weeks after treatment cessation (Fig. 4*A*), and EFdA plus AUY922 treatment exerted the same rebound-suppressing effect. No adverse events were observed in any of the treated mice. Improved second-generation clinical Hsp90 inhibitors, such as 17-AAG and AUY922, display minimal toxicity in nonmalignant cells and are generally well tolerated in small animal models (24). One *in vitro* study suggested that geldanamycin (the first-generation parent compound of 17-AAG) down-regulated T-lymphocyte cell surface antigens and receptors (61). As observed with the previous experimental cohort, the mouse peripheral blood human CD4^+^ T cell counts at the time of treatment cessation were highly similar between the untreated, 17-AAG-treated and AUY922-treated groups. In addition, no signs of toxicity or weight loss were seen in the mice after 2 weeks of treatment with either 17-AAG or AUY922 at the dosage level we used (10 mg/kg/day). The long term suppression of plasma viremia may be attributed to the anti-inflammatory effect of 17-AAG and AUY922 (27). Hsp90 inhibitors have been shown to reduce inflammation, and increased viremia in HIV-infected patients is often observed in the presence of chronic inflammation (2, 4).

**FIGURE 5.**
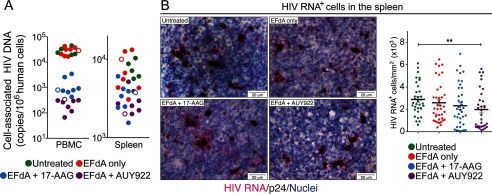
**Dramatically reduced HIV DNA copy number in circulating PBMC from Hsp90 inhibitor-treated mice yet minimal reduction of HIV DNA in spleen cells with decreased numbers of HIV RNA-positive cells.** *A*, cell-associated HIV DNA copy number was estimated on total DNA extracted from NSG-BLT mouse PBMC and spleen cells. *Open circles*, mice that were not injected with CD34^+^ hematopoietic stem/progenitor cells. The HIV DNA copy number is compared with that of human CCR5 DNA using PCR primers specific to human CCR5. *B*, HIV RNA (coding RNA^+^, *fuchsia*) was detected by RNAscope using a HIV gag-pol probe in formaldehyde-fixed frozen spleen tissue samples (obtained 11 weeks after treatment cessation from mice shown in *A*). The RNAscope assay was followed by colorimetric immunohistochemistry for p24 (*brown*), and nuclei were counterstained with hematoxylin. Images were acquired at ×630 magnification, and HIV RNA-positive cells were digitally quantified by ImageJ software analysis. The total number of HIV RNA-positive cells (abundantly expressing HIV RNA or containing a single HIV RNA transcript) was quantified using 34 images from three spleens (untreated group), 38 images from four spleens (EFdA only), 37 images from four spleens (EFdA plus 17-AAG), and 33 images from four spleens (EFdA plus AUY922). In the HIV RNA^+^ bar graph, *p* = 0.0071 (**) by Mann-Whitney *U* test.

The lack of plasma HIV RNA rebound was corroborated by IUPM assays with peripheral blood cells; 6 of 8 mice treated with 17-AAG and all 8 mice treated with AUY922 had no detectable infectious HIV 6 and 11 weeks after treatment cessation (Fig. 4*B*). Low IUPM levels (200–1,000) were detected, however, in spleen cells from the Hsp90 inhibitor-treated mice, indicating the presence of a tissue reservoir of persistent infection in the nonviremic mice. Most importantly, we were able to recover infectious HIV from PBMC at 39.5 °C, and we observed enhanced virus titers when the PBMC and spleen cells were activated with SAHA (62), highlighting the existence of a replication-competent HIV reservoir that can be suppressed by Hsp90 inhibition. AUY922 appeared to be more effective than 17-AAG in reducing HIV plasma viremia as evidenced by the lower HIV DNA copy numbers in the PBMC of AUY922-treated mice (Fig. 5*A*). However, we observed comparable HIV DNA copy numbers between the untreated and Hsp90 inhibitor-treated groups (Fig. 5*A*). We confirmed the presence of this replication-competent reservoir in the spleen by RNAscope *in situ* hybridization (Fig. 5*B*), and AUY922 appears to significantly reduce HIV RNA transcription in the tissue reservoir. The comparable integrated HIV DNA copy numbers between the untreated and Hsp90 inhibitor-treated groups suggest that persistent tissue reservoirs are established in the mice soon after HIV exposure, and the presence of active HIV RNA transcription in the untreated mice indicates that infected spleen cells are the likely source of plasma viremia after antiretroviral therapy cessation. Hsp90 inhibitors are not expected to purge integrated HIV; rather, the mechanism of action of 17-AAG and AUY922 is suppression of HIV transcription, which is made evident by lower RNA copies in the spleen of AUY922-treated mice. These findings demonstrate that tissue reservoirs produce replication-competent virus that, in the absence of Hsp90 inhibition, readily replenishes plasma viremia after EFdA cessation (Fig. 4*A*, *EFdA only*). Hsp90 inhibition thus prevented viremia rebound and reduced the spleen virus reservoir, most likely by silencing HIV transcription and blocking virus reactivation.

##### Heat Shock Accelerates HIV Transcription and Virion Production

**FIGURE 6.**
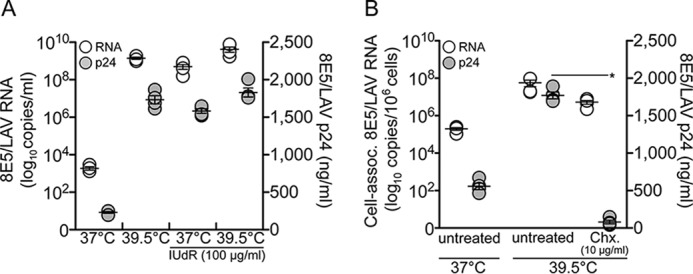
**Heat shock (39.5 °C) accelerates HIV transcription in persistently infected cells.** *A*, virion RNA and p24 levels in culture supernatants from 8E5/LAV cells at 37 and 39.5 °C and in the presence of 100 μg/ml IUdR. *p* < 0.02 comparing 8E5/LAV RNA copy number at 37 °C with 8E5/LAV RNA copy number at 39.5 °C, 37 °C + IUdR, and 39.5 °C + IUdR by Student's *t* test. *p* < 0.0001 comparing 8E5/LAV p24 at 37 °C with 8E5/LAV p24 at 39.5 °C, 37 °C + IUdR, and 39.5 °C + IUdR by Student's *t* test. *B*, cell-associated HIV RNA and supernatant p24 levels from 8E5/LAV cells treated at 39.5 °C with cycloheximide (*Chx*). *, *p* < 0.0001, comparing 8E5/LAV p24 at 39.5 °C with 8E5/LAV p24 at 39.5 °C in the presence of cycloheximide. Data in both graphs represent mean ± S.E. (*error bars*) (*n* = 4).

The effect of heat shock on HIV transcription has been described; however, previous studies by others and by us have not studied the effect of 39.5 °C beyond HIV transcription. Heat shock conditions could potentially cause *de novo* virus production, so to confirm our observations that Hsp90 inhibitors blocked HIV transcription, we investigated the global effect of 39.5 °C and Hsp90 inhibition in chronically infected cells. To determine the specific effects of 39.5 °C on HIV transcription *in vitro*, we selected the chronically infected 8E5/LAV cell line, which contains a single full-length integrated HIV genome (55). 8E5/LAV cells can be stimulated with the halogenated pyrimidine IUdR to produce mature virions that are noninfectious because of a point mutation in reverse transcriptase (RT). The key advantage of this assay system is that virion production is directly proportional to HIV gene expression and is independent of virus spread (63). 8E5/LAV cells were cultured in the presence of IUdR at 39.5 °C for 48 h, and the culture supernatant was collected for quantification of both HIV RNA (by quantitative PCR) and p24 antigen (Fig. 6*A*). Interestingly, 39.5 °C alone increased HIV RNA and p24 antigen levels (10^9.1^ HIV RNA copies and 1700 ng of p24/ml) more than IUdR stimulation (10^8.7^ HIV RNA copies and 1600 ng of p24/ml) at 37 °C. A further, but marginal, increase was observed when 8E5/LAV cells were incubated with IUdR at 39.5 °C (10^9.6^ HIV RNA copies and 1800 ng of p24/ml), suggesting that the elevated temperature alone could saturate HIV RNA synthesis and virus production. To confirm that virion production at 39.5 °C resulted from accelerated HIV transcription, 8E5/LAV cells were tested in the presence of cycloheximide (Fig. 6*B*) to selectively inhibit eukaryotic protein biosynthesis (64). Cycloheximide did not affect cell-associated HIV RNA levels but completely blocked virion production (*p* < 0.0001), confirming that 39.5 °C accelerates viral RNA transcription.

We confirmed that assay conditions at 39.5 °C and the presence of IUdR did not affect the integrity of HIV by Western blotting (data not shown). Further, we confirmed that 39.5 °C did not rescue the noninfectious 8E5/LAV phenotype, and virus harvested from culture supernatant at 39.5 °C was RT-deficient and remained noninfectious (data not shown). Assay conditions at 39.5 °C did not increase 8E5/LAV cell numbers or affect cell viability, and we confirmed that incubation at 39.5 °C for 48 h did not alter cellularity using a sensitive quantitative acetylcholinesterase assay (*V*_max_ at 37 °C = 2.38 and *V*_max_ at 39.5 °C = 2.53) and by quantitative fluorescence Western blotting of two housekeeping genes (α-GAPDH and α-actin; data not shown).

##### Increased Hsp90 Activity at 39.5 °C Accelerates HIV Transcription

**FIGURE 7.**
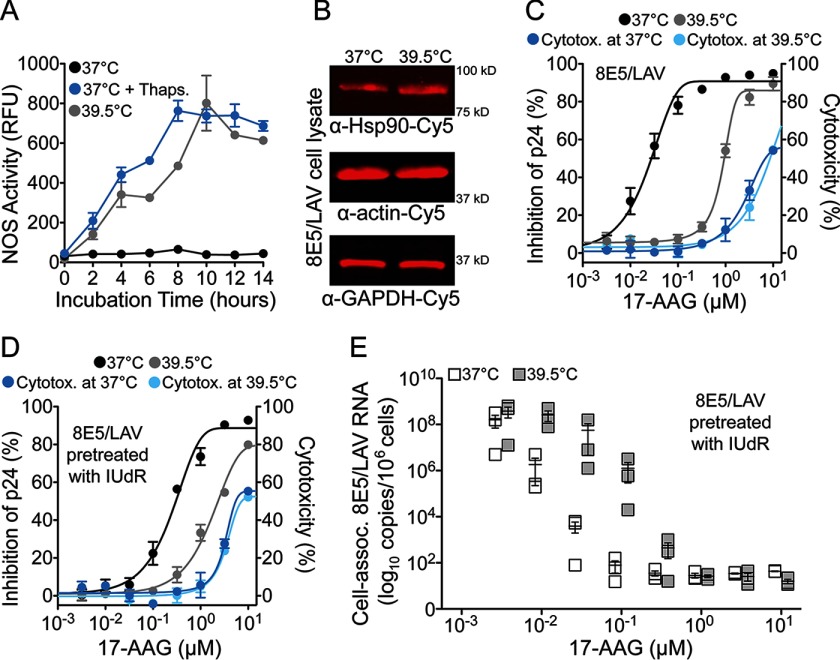
**Increased cellular Hsp90 activity accelerates HIV transcription in persistently infected cells.** *A*, nitric-oxide synthase (*NOS*) activity in 8E5/LAV cells at 37 and 39.5 °C with thapsigargin (*Thaps*.) as a positive control at 37 °C. Data represent mean ± S.E. (*n* = 3). *B*, quantitative fluorescence Western blotting analysis of 8E5/LAV cell lysates. Results shown are representative of four independent experiments. *C*, antiviral activity of 17-AAG in 8E5/LAV cells at 37 and 39.5 °C. Mean cytotoxicity at 37 °C (5.2 μm) and 39.5 °C (5.2 μm) is shown. *D*, antiviral activity of 17-AAG on 8E5/LAV cells pretreated with IUdR at 37 and 39.5 °C. Mean cytotoxicity at 37 °C (5.6 μm) and 39.5 °C (5.3 μm) is shown. *E*, cell-associated HIV RNA from 8E5/LAV cells pretreated with IUdR at 37 and 39.5 °C. *p* = 0.26 at 100 nm 17-AAG and *p* = 0.34 at 32 nm 17-AAG by Student's *t* test. Data represent mean ± S.E. (*error bars*) (*n* = 3).

To determine whether accelerated HIV transcription at 39.5 °C is mediated by increased Hsp90 activity, we assayed nitric-oxide synthase activity as an indicator of Hsp90 activation (47) and found that at 39.5 °C, Hsp90 activity increased >700-fold (*p* = 0.0016) in 8E5/LAV cells (Fig. 7*A*) with a ∼2-fold increase in Hsp90 protein expression (Fig. 7*B*). To determine the role of Hsp90 in heat shock-mediated acceleration of HIV production, we treated 8E5/LAV cells with 17-AAG, which inhibited HIV production (IC_50_ = 0.026 μm) at 37 °C, but at 39.5 °C, the drug was 40-fold less potent (IC_50_ = 0.95 μm, *p* < 0.0001) (Fig. 7*C*). 8E5/LAV cells were then stimulated with IUdR, and HIV production was measured in the presence of 17-AAG (Fig. 7*D*). As in the absence of IUdR, we observed potent inhibition of HIV production at 37 °C (IC_50_ = 0.28 μm) but significantly less antiviral activity at 39.5 °C (IC_50_ = 2.3 μm, *p* = 0.0003). In support of our hypothesis, inhibition of Hsp90 with 17-AAG resulted in a dose-dependent decrease in HIV transcription at 37 °C (Fig. 7*E*). In contrast, HIV transcription at 39.5 °C proceeded within the inhibitory range of 17-AAG (10^−1^ to 10^−2^ μm). Our results indicate that increased Hsp90 activity (∼750-fold; Fig. 7*A*) at 39.5 °C supported HIV transcription by saturating the antiviral effect of 17-AAG. To rule out the possibility that 17-AAG was biologically inactive at 39.5 °C, cell lysates from 8E5/LAV cells treated with 17-AAG at 39.5 °C were incubated with heat-denatured firefly luciferase in a Hsp90-dependent protein refolding assay (48). Lysates from cells treated with 17-AAG at 39.5 °C with ≤10^−1^ μm 17-AAG exhibited dose-dependent luciferase activity, most likely due to an increase in Hsp90 activity at 39.5 °C. Nevertheless, cell lysates from 8E5/LAV cells treated with ≥1 μm 17-AAG at 37 and 39.5 °C failed to renature the inactive heat-denatured luciferase to the same extent, thus confirming that 17-AAG inhibited Hsp90 at 39.5 °C (data not shown).

##### Increased HIV Production at 39.5 °C Resulted Directly from Accelerated Viral Transcription

**FIGURE 8.**
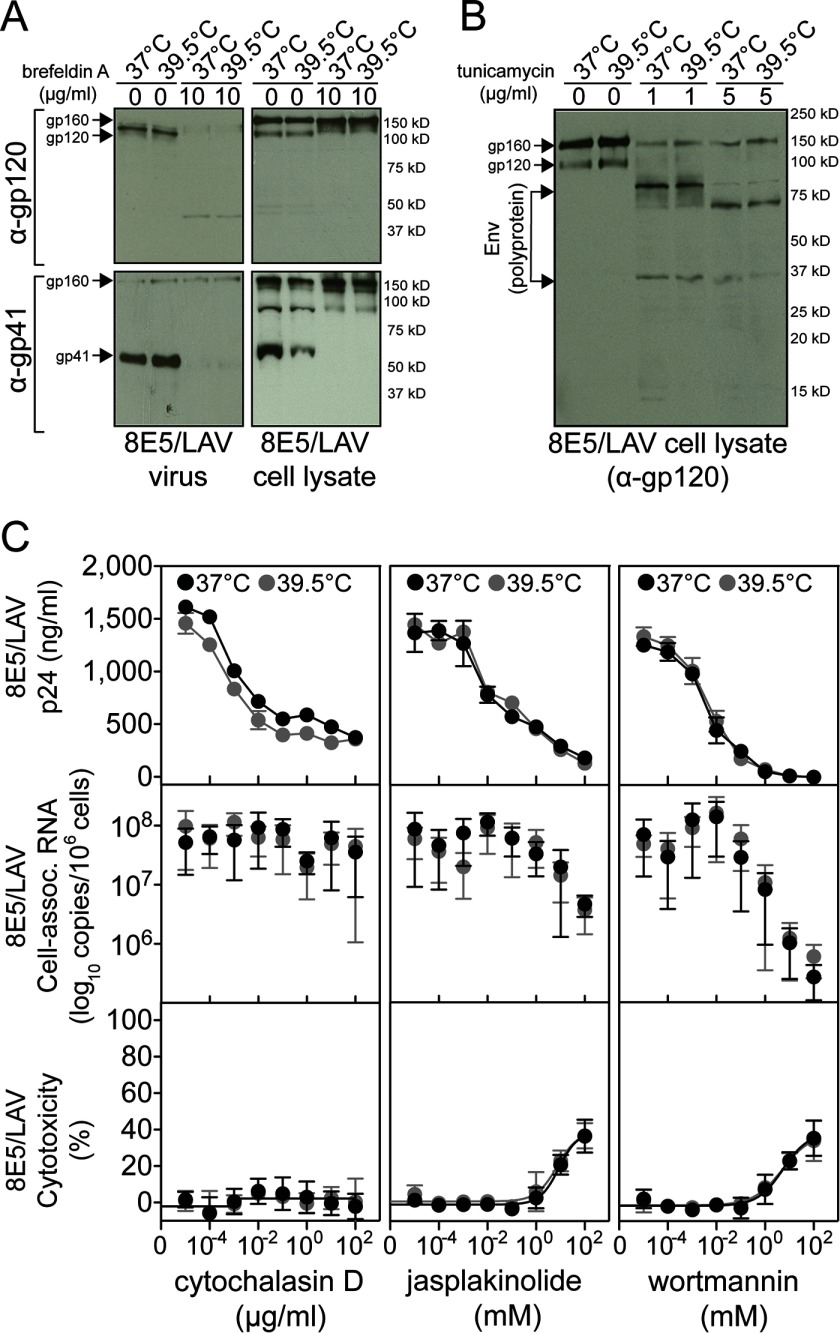
**Processing of 8E5/LAV envelope glycoprotein and intracellular transport and release of mature virions at 39.5 °C.** Actively growing 8E5/LAV cells were divided evenly and incubated at 37 °C with 100 μg/ml IUdR or at 39.5 °C in the presence of the following agents. *A*, 8E5/LAV culture treated with brefeldin A was assayed for supernatant 8E5/LAV virus and cell lysates for viral gp120 and gp41 by immunoblotting. Results shown are representative of four independent experiments. *B*, 8E5/LAV culture treated with tunicamycin was assayed for intracellular viral gp120 by immunoblotting. Results shown are representative of four independent experiments. *C*, 8E5/LAV culture in the presence of cytochalasin D, jasplakinolide, and wortmannin, at the indicated drug concentration. Culture supernatants were assayed for HIV p24 protein, cell lysates were assayed for cell-associated 8E5/LAV RNA copy number by quantitative PCR, and cytotoxicity of the test agents was determined by the MTT assay. Data represent mean ± S.E. (*error bars*) (*n* = 3).

To determine whether 39.5 °C could enhance virion production above the observed increase in HIV RNA transcription, we assayed a succession of pharmacologic agents previously known to inhibit specific stages of HIV maturation and release of infectious virions. For each test agent, an actively growing 8E5/LAV cell culture was first washed in PBS to remove residual virions attached to the cell membrane and resuspended in fresh medium to ensure that all viral RNA or p24 detected was from newly synthesized virions, and then an equal number of cells were incubated at 37 °C with IUdR and at 39.5 °C for 6 h to a maximum of 8 h because of the inherent toxicity of the test agents. Further multiple concentrations were assayed, and dose-response curves were generated because subtle phenotypic changes in virion production may not be observed at any one given concentration. In addition, incubation times were standardized for each agent to ensure that the effect on viral RNA and p24 was not a consequence of general toxicity and cell cycle arrest. The exclusive effect of 39.5 °C on HIV transcription was further shown by normal virion production after treatment with brefeldin A (65), tunicamycin A (66), cytochalasin D (67), jasplakinolide (68), and wortmannin (67). Treatment with brefeldin A at 39.5 °C (Fig. 8*A*) resulted in typical virus-like particles that lack the gp41 and gp120 envelope subunits (69). Similarly, treatment with tunicamycin A indicated that 39.5 °C did not compensate for HIV envelope maturation (Fig. 8*B*). Cytochalasin D and jasplakinolide are known to interfere with intracellular transport of HIV proteins, and wortmannin prevents HIV budding from the cell membrane. In the absence of cytotoxicity and in a dose-dependent manner, these compounds revealed normal intracellular transport and release of mature HIV virions at 39.5 °C (Fig. 8*C*). Current knowledge indicates that Hsp90 facilitates multiple stages in the life cycle of numerous viruses, and our earlier work emphasized the importance of Hsp90 in HIV infectivity (21). Here, we intentionally used a model of HIV persistence wherein Hsp90-mediated HIV transcription can be uncoupled from subsequent effects of Hsp90 on virus maturation and infectivity. Our results thus indicate that 39.5 °C specifically accelerates HIV RNA transcription by Hsp90-mediated activation of host transcription factors.

**FIGURE 9.**
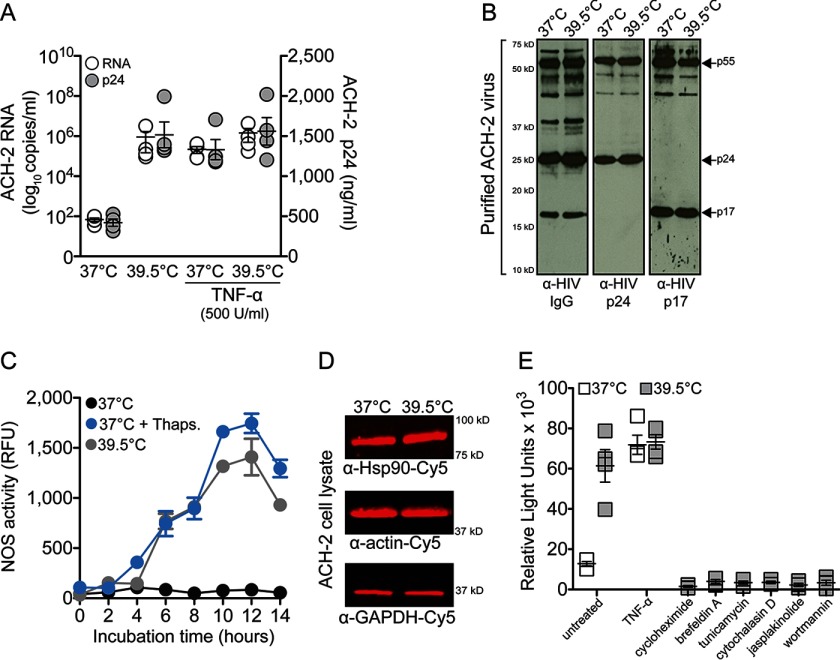
**Heat shock (39.5 °C) accelerates HIV transcription in persistently infected ACH-2 cells.** *A*, HIV RNA and p24 levels in culture supernatants from ACH-2 cells incubated at 37 and 39.5 °C. TNF-α was used to activate HIV transcription at 37 °C. *p* < 0.001 comparing ACH-2 p24 at 37 °C with ACH-2 p24 at 39.5 °C, 37 °C + TNF-α, and 39.5 °C + TNF-α by Student's *t* test. Data represent mean ± S.E. (*n* = 4). *B*, Western blotting analysis of the purified ACH-2 virions. *C*, nitric-oxide synthase activity in ACH-2 cells at 37 and 39.5 °C; thapsigargin was used as a positive control at 37 °C. Results represent mean ± S.E. (*error bars*) (*n* = 3). *D*, quantitative fluorescence Western blotting analysis of ACH-2 cell lysates. Results are representative of four independent experiments. *E*, TZM-bl luciferase assay of TZM-bl cells inoculated with an equal volume of culture supernatants from ACH-2 cells treated with the indicated agents. *p* = 0.001 comparing ACH-2 infectivity at 37 °C with ACH-2 infectivity at 39.5 °C by Student's *t* test. Data represent mean ± S.E. (*n* = 3).

To determine the effect of heat shock and increased Hsp90 activity on HIV infectivity, we selected the chronically infected ACH-2 cell that can be activated by TNF-α to produce infectious virions (70). Heat shock was sufficient to accelerate HIV transcription and virion production comparable with TNF-α treatment (Fig. 9*A*), and as we observed before, 39.5 °C did not affect the virus phenotype (Fig. 9*B*). Hsp90 activity increased by ∼1,500-fold (*p* = 0.0003) (Fig. 9*C*) with ∼1.5-fold increased protein expression (Fig. 9*D*) at 39.5 °C, and HIV virions were highly infectious, comparable with infectious virus obtained with TNF-α activation (Fig. 9*E*). Infectious virus production at 39.5 °C was inhibited by the aforementioned cytostatic agents, confirming that increased Hsp90 activity specifically accelerates HIV transcription.

## Discussion

Initial evidence that integrated provirus is silent in persistent HIV reservoirs came from a subset of transformed cells that survived infection with low or absent HIV gene expression. Subsequently, it was established that genomic HIV RNA synthesis is stimulated by inducible host transcription factors that are only transiently active and that HIV gene expression is silenced when the cell reverts back to the resting stage in the absence of cellular activation. Here we report that increased Hsp90 activity in activated cells is necessary for HIV transcription and that Hsp90 inhibitors prevent rebound viremia *in vivo*.

Cellular activation results in increased transcription, and the Hsp90 molecular chaperone is primarily responsible for transient activation of inducible host transcription factors that translocate to the nucleus and cause gene expression. To demonstrate the effect of Hsp90 on HIV transcription, we determined the effect of heat shock on gene expression from the HIV promoter and by individual host transcription factors. Transcription from the intact HIV promoter was greatly enhanced by heat shock, and Hsp90 inhibition dramatically reduced gene expression. Similarly, transcription by the NF-κB-, NFAT-, and STAT5-inducible transcription factors increased severalfold with heat shock, and Hsp90 inhibition significantly reduced gene expression. Interestingly, the constitutive AP1 host transcription factor, also essential for HIV transcription, was unaffected by heat shock, and AP1 transactivated gene expression despite Hsp90 inhibition. Our observations indicate that HIV transcription is regulated by Hsp90 and imply that persistent HIV reservoirs are established because of the absence of Hsp90-activated host transcription factors in resting cells.

Transcriptionally silent persistent HIV reservoirs exist in tissues and are the source of rebound viremia after ARV cessation. Based on the above observations, we reasoned that Hsp90 inhibition should prevent rebound viremia after ARV cessation and thus tested the antiviral effects of Hsp90 inhibition in an *in vivo* model of HIV infection. Robust HIV infection in humanized mice was reduced to undetectable levels in the plasma by EFdA, an ultrapotent reverse transcriptase inhibitor. Mice were treated with 17-AAG either concurrently with EFdA or after EFdA treatment. Three weeks after therapy cessation, a majority of the mice had no evidence of rebound viremia, and even after 7 weeks after treatment cessation, one-third of the mice had no detectable plasma HIV RNA. These observations indicate a reduction in the reservoir of persistently infected cells during 17-AAG treatment because mouse peripheral blood human CD4^+^ T cell counts at the time of treatment cessation were highly similar between groups. Hence, the lack of viral rebound in the 17-AAG-treated mice was not the result of drug-mediated loss of HIV target cells. The lack of persistently infected cells in the peripheral blood of 17-AAG-treated mice was confirmed by a sensitive viral outgrowth assay, and in the EFdA plus 17-AAG-treated group, we detected replication-competent virus in only one mouse 3 weeks after treatment cessation.

In the second humanized mouse study, we sought to further characterize the effect of Hsp90 inhibition on persistent HIV infection and included a more potent Hsp90 inhibitor. Similar to the previous mouse cohort, concurrent treatment with EFdA and the Hsp90 inhibitor resulted in undetectable plasma HIV RNA up to 11 weeks after treatment cessation. Hsp90 inhibitor treatment greatly reduced the HIV DNA copy number in the peripheral blood, and replication-competent virus was not detected in most of the EFdA plus 17-AAG-treated mice and all of the EFdA plus AUY922-treated mice. Nevertheless, cellular activation by heat shock and SAHA resulted in detectable replication-competent virus, suggesting that Hsp90 inhibition prevented HIV transcription in persistently infected cells. These results further indicate that cellular activation is necessary to reactivate HIV transcription and explain why the mouse plasma HIV RNA levels remained undetectable long after treatment cessation. At this time, we are uncertain whether these persistently infected cells preexisted in the peripheral circulation or if they migrated from a tissue reservoir.

Conversely, replication-competent virus was readily recovered from the spleens of Hsp90 inhibitor-treated mice, indicating that a tissue reservoir in the mouse spleen is established soon after exposure to HIV. Interestingly, the HIV DNA copy number in the spleen was uniform between the treated and untreated groups, but the number of human cells expressing viral RNA was significantly reduced in the AUY922-treated mice. These results suggest that Hsp90 inhibition also reduced HIV transcription in the spleen reservoir because heat shock and SAHA increased the recovery of replication-competent virus. These *in vivo* observations provide convincing evidence that replication-competent tissue reservoirs are the source of viremia after antiretroviral therapy cessation and that Hsp90 inhibition can suppress HIV transcription in persistently infected cells, thus preventing rebound in viremia.

The chronically infected 8E5/LAV and ACH-2 cell lines have been used historically to characterize persistent HIV infection, and we observed that heat shock independently accelerated HIV transcription comparable with that by cell-activating agents. Heat shock specifically increased HIV transcription, and inhibition of the host transcriptome prevented viral RNA synthesis. Blocking cellular protein synthesis did not affect accelerated HIV transcription but prevented virion production, indicating that the effect of heat shock was analogous to that of cell-activating agents. We previously demonstrated that Hsp90 inhibition has a potent antiviral effect and now show that heat shock can sustain HIV transcription in the presence of Hsp90 inhibitors because of increased Hsp90 activity and intracellular expression. Detailed analysis revealed that increased virion production by heat shock directly resulted from accelerated HIV transcription and that the elevated temperature did not affect virus maturation or release.

In conclusion, our data indicate that Hsp90 activity is necessary to reactivate HIV transcription in persistent virus reservoirs. Current knowledge indicates that in activated cells, Hsp90 transiently activates inducible host transcription factors required for HIV transcription; however, HIV gene expression is silenced when the cell reverts back to a resting state. Targeting HIV reactivation might prevent rebound in viremia after ARV cessation, and including Hsp90 inhibitors in current therapy regimens could lead to long term remission and possibly a functional cure if rebound can be prevented indefinitely.

## Author Contributions

P. J. and C. A. S. conceived and designed the study. E. M. performed the RNAScope analysis. P. J. and C. A. S. wrote the manuscript.

## Footnotes

2 The abbreviations used are:

Hsp90: heat shock protein 90
SAHA: suberoylanilide hydroxamic acid
PBMC: peripheral blood mononuclear cell(s)
PHA: phytohemagglutinin
NSG-BLT: NOD-scid IL-2Rγ^−/−^ bone marrow-liver-thymus
IUdR: 5′-iodo-2′-deoxyuridine
EFdA: 4′-ethynyl-2-fluoro-2′-deoxyadenosine
17-AAG: 17-(allylamino)-17-demethoxygeldanamycin (tanespimycin)
AUY922: NVP-AUY922
IUPM: infectious units per million cells
MTT: 3-(4,5-dimethylthiazol-2-yl)-2,5-diphenyl-tetrazolium bromide
CC_50_: 50% reduction in cell viability.
